# Understanding the mechanisms of efficacy of fecal microbiota transplant in treating recurrent *Clostridioides difficile* infection and beyond: the contribution of gut microbial-derived metabolites

**DOI:** 10.1080/19490976.2020.1810531

**Published:** 2020-09-06

**Authors:** Laura Martinez-Gili, Julie a K McDonald, Zhigang Liu, Dina Kao, Jessica R Allegretti, Tanya M Monaghan, Grace F Barker, Jesús Miguéns Blanco, Horace R T Williams, Elaine Holmes, Mark R Thursz, Julian R Marchesi, Benjamin H Mullish

**Affiliations:** a Division of Systems Medicine, Department of Metabolism, Digestion and Reproduction, Faculty of Medicine, Imperial College London, London, UK; b MRC Centre for Molecular Bacteriology and Infection, Imperial College London, London, UK; c Division of Digestive Diseases, Department of Metabolism, Digestion and Reproduction, Faculty of Medicine, Imperial College London, London, UK; d Division of Gastroenterology, Department of Medicine, University of Alberta, Edmonton, Alberta, Canada; e Division of Gastroenterology, Hepatology and Endoscopy, Brigham and Women’s Hospital, Boston, MA, USA; f Harvard Medical School, Harvard University, Boston, MA, USA; g NIHR Nottingham Biomedical Research Centre, University of Nottingham, Nottingham, UK; h Nottingham Digestive Diseases Centre, School of Medicine, University of Nottingham, Nottingham, UK; i Institute of Health Futures, Murdoch University, Perth, Western Australia; j School of Biosciences, Cardiff University, Cardiff, UK

**Keywords:** Gut microbiome, metabonomics, fecal microbiota transplant, *Clostridioides difficile* infection, bile acids, short chain fatty acids, trimethylamine

## Abstract

Fecal microbiota transplant (FMT) is a highly-effective therapy for recurrent *Clostridioides difficile* infection (rCDI), and shows promise for certain non-CDI indications. However, at present, its mechanisms of efficacy have remained poorly understood. Recent studies by our laboratory have noted the particular key importance of restoration of gut microbe-metabolite interactions in the ability of FMT to treat rCDI, including the impact of FMT upon short chain fatty acid (SCFAs) and bile acid metabolism. This includes a significant impact of these metabolites upon the life cycle of *C. difficile* directly, along with potential postulated additional benefits, including effects upon host immune response. In this *Addendum*, we first present an overview of these recent advancements in this field, and then describe additional novel data from our laboratory on the impact of FMT for rCDI upon several gut microbial-derived metabolites which had not previously been implicated as being of relevance.

## 1. Introduction

Fecal microbiota transplant (FMT) is widely-recognized as a highly-efficacious treatment for recurrent/refractory *Clostridioides difficile* infection (rCDI) that has not responded to conventional pharmacological therapy, such as fidaxomicin or vancomycin.^1,2^ There is also growing interest in the potential application of FMT for a range of non-CDI indications.^3^ However, there are certain clear drawbacks related to the use of FMT in its present form, including the complex regulation associated with its use, the potential need for invasive administration (i.e. endoscopy – although capsulized FMT is increasingly available), and the risk of transmission of infection from donor to recipient. The latter point is particularly salient, given a recent report of multi-drug resistant *E. coli* being transmitted via FMT (to an immunosuppressed patient without rCDI) with a resultant patient death.^4^ Furthermore, there are currently no established biological means for matching stool donor with recipient, despite certain proposals.^5^ As such, deconvoluting the mechanisms of efficacy of FMT – and exploiting this knowledge to develop novel targeted microbiome therapeutics, or to better match donor and recipient – is a major clinical priority.

FMT for rCDI rapidly restores a gut microbiota, that has been hugely disrupted by recurrent antibiotic therapy, back toward pre-morbid composition and diversity, resembling that of healthy donors.^6,7^ Proof-of-concept studies have further inferred the central role of restoration of gut bacteria specifically in the efficacy of FMT, demonstrating that either a healthy donor-derived defined community of commensal bacteria^8^ or fractionated spores from ethanol-shocked donor stool^9^ may have comparable efficacy to conventional FMT in treating rCDI. However, a more recent pilot study demonstrated that sterile filtered donor stool (filtered through a 0.2 μm filter, small than the size of a bacterium) also effectively caused sustained remission from rCDI.^10^ This study raised the intriguing possibility that soluble factors – rather than intact bacteria *per se –* were key mediators for the efficacy of FMT.

Among such possible factors, non-bacterial gut microbiota components have been one area of focus. Several studies have described that the stool donor virome or mycobiome is associated with the efficacy of FMT for rCDI, and that these undergo rapid changes in the stool of rCDI patients treated successfully with FMT.^11–14^ However, the significance of these changes as a potential mechanism of efficacy of FMT remains unclear. For example, given the narrow range that bacteriophages possess, any anti-*C. difficile* lytic phages in the gut virome post-FMT would have had to have originated from *C. difficile* in the donor virome; given that *C. difficile* carriage is a near universal exclusion criterion for acceptance as an FMT donor, this would be very unlikely in practice. Similarly, given the established relationship between antimicrobial treatment and *Candida* overgrowth within the gut, any changes in gut mycobiome profiles post-FMT might only represent proxies of gut bacterial alterations. Consequently, the specific contribution of bacteriophages and fungi to the efficacy of FMT remains undefined.

A further key area of interest has been whether a potential mechanism of FMT may be through the restoration of microbial metabolites, or of co-metabolites derived from interaction between the gut microbiota and host. This hypothesis has been the focus of much recent research from our laboratory. In the coming sections, we first summarize our recently-published work in this area, before introducing additional novel data.

## 2. Impact of FMT for rCDI upon gut microbial metabolites: recently-discovered areas

### 2.1. Bile acid metabolism

Different bile acids have varied effects upon the ability of *C. difficile* to undergo germination or vegetative growth *in vitro*. Specifically, the conjugated primary bile taurocholic acid (TCA) promotes spore germination of *C. difficile* (with glycine functioning as pro-germinant);^15,16^ in contrast, secondary bile acids (including deoxycholic acid (DCA)) inhibit the vegetative growth and toxin activity of the bacterium.^15,17^ In mammals, conversion from primary to secondary bile acids occurs within the distal gut, undertaken by several enzymes produced by the gut microbiota, but not by mammals. The two principle enzymatic processes are a first step mediated by *bile salt hydrolase (BSH*; which hydrolyzes the glycine or taurine group from conjugated bile acids, aka choloylglycine hydrolase (EC 3.5.1.24)) and a second step by 7-α-dehydroxylase (which converts unconjugated primary bile acids into secondary bile acids).^18,19^ Recent studies demonstrated that the activity of bile-metabolizing enzymes (7-α-dehydroxylase in particular) is partly protective against CDI in rodents.^20,21^ Therefore, one hypothesis has been as to whether patients with rCDI – with antibiotic-mediated destruction of their gut microbiota – are deficient in gut microbiota members which produce bile-metabolizing enzymes, with the consequent enrichment in TCA (promoting *C. difficile* germination) and loss of DCA (facilitating vegetative growth) perpetuating active disease. By extension, FMT may restore bacteria that produce these enzymes, and reverse the abnormal bile acid *milieu* of the distal gut.

Supporting this hypothesis, the stool bile acid *milieu* is enriched in TCA in human patients with rCDI, whilst secondary bile acids predominate in post-FMT stool.^22–25^ Similarly, healthy donor stool contains little TCA, but relatively high levels of secondary bile acids.^22–24^ Further recent work from our laboratories explored the dynamics of microbial bile acid-metabolizing enzymes in patients with rCDI, and the impact of FMT upon this. Predicted *bsh* gene abundance was significantly reduced in patients with rCDI, compared to patients with a primary episode of CDI, and/or healthy controls.^24^ Furthermore, the stool microbiota from patients pre-FMT had a greatly reduced relative abundance of a broad range of BSH-producing bacteria compared to the stool of patients treated successfully with FMT and/or healthy donors.^23^ Successful FMT for rCDI rapidly and sustainably restored stool *bsh* gene copy number and BSH functional activity from the almost negligible levels found pre-FMT up to comparably high levels to that found in healthy donors.^23^ Finally, stool *C. difficile* counts were ~70% reduced in an rCDI mouse model after administration of *E. coli* expressing highly-active BSH relative to mice administered BSH-negative *E. coli*.^23^ Collectively, these data are strongly supportive that FMT-mediated restoration of gut microbial bile acid metabolism – and particularly BSH functionality – is a key mechanism underlying FMT’s efficacy in rCDI. An additional recent relevant finding has been that gut bacteria expressing 7-α-dehydroxylase are also able to produce tryptophan-derived antibiotics, which themselves inhibit the cell division of *C. difficile*.^26^

In addition to the direct action of bile acids upon *C. difficile*, recent findings hint at complementary mechanisms by which gut microbiota-bile acid interactions contribute to protection from CDI. For example, successful FMT for rCDI was also found to be associated with increased circulating fibroblast growth factor (FGF)-19, consistent with the upregulation of the farnesoid X receptor (FXR)-FGF pathway.^27^ In line with this, pre-treatment with the tertiary bile acid ursodeoxycholic acid (UDCA) in a CDI mouse model was associated with increased FXR-FGF signaling-related transcripts and attenuated inflammation.^28^ Furthermore, upregulated FXR after FMT for rCDI may contribute to the resolution of the colitis induced by *C. difficile,* since FXR agonist administration in a rodent colitis model was associated with reduced colonic inflammation and a more intact intestinal barrier.^29^ Ileal FXR activation also causes reduced hepatic bile acid synthesis by negative feedback; this may result in reduced TCA secretion into the gut, further reducing germination of *C. difficile*. Additionally, microbially-mediated production of certain secondary bile acids has been demonstrated to promote the generation of peripheral regulatory T cells,^30^ linking these metabolites with colonic immunity.

### 2.2. Short chain fatty acid metabolism

One further group of metabolites well-studied in this field are the short chain fatty acids (SCFAs). The major source of these is bacterial fermentation of partially and non-digestible carbohydrates (primarily dietary), although certain amino acids may also be a source.^31^ In rodent studies, while antibiotics reduced SCFA levels in stool, higher SCFA levels were found to be associated with protection from *C. difficile* growth, suggesting an interplay between antibiotics, SCFAs, and rCDI risk.^17^

Human studies have demonstrated that levels of a range of SCFAs within stool (including acetate, propionate and butyrate) are very low in rCDI, but restored to levels comparable to healthy donors after successful FMT.^25^ However, given the close link between antibiotic use, dietary intake and SCFA production, it is difficult to ascertain from human observational studies alone whether the post-FMT increase in stool SCFAs reflects changes in dietary intake and/or recovery after antibiotic discontinuation post-resolution of CDI, or whether this is driven by specific FMT-related gut microbiota changes.

As such, our laboratory investigated this using an artificial gut (“chemostat”) model of CDI, whereby the confounding factor of variable dietary intake was removed.^32^ In these experiments, recovery of the levels of certain SCFAs (including butyrate) was observed after cessation of antibiotics and even prior to FMT, consistent with spontaneous gut microbiota recovery. However, levels of valerate (the five carbon SCFA) only recovered to baseline levels after the administration of FMT, and not after antibiotic cessation alone. Valerate caused a dose-dependent inhibition of the vegetative growth of several *C. difficile* ribotypes, but no adverse growth effects upon several different common gut commensal bacteria. In a CDI mouse model, oral administration of valerate (in the form of glycerol trivalerate) resulted in a ~ 95% reduction in *C. difficile* stool counts compared to control-treated mice.^32^ Additionally, successful FMT for rCDI in humans was associated with rapid, sustained restoration of stool valerate to similar levels as found in stool donors.^32^

Restoration of SCFAs by FMT may benefit patients recovering from CDI by mechanisms beyond inhibiting the growth of *C. difficile*, including a possible role in the resolution of colitis and gut barrier function. For instance, in a mouse model of CDI, butyrate was demonstrated to improve intestinal barrier function and reduce intestinal inflammation via a mechanism involving activation of the transcription factor HIF-1.^33^ Furthermore, SCFAs regulate the size and function of the colonic regulatory T cell population in mice, which has been directly demonstrated to be a protective mechanism against colitis.^34^ SCFAs (including valerate) are also recognized to inhibit histone deacetylases (HDACs), with the resultant change in transcription of a range of genes having a net anti-inflammatory effect on the host immune phenotype.^35,36^ Of particular relevance, exogenous valerate has already been shown to ameliorate chemically-induced colitis in a rodent model via HDAC inhibition.^37^

## 3. Impact of FMT for rCDI upon gut microbial metabolites: novel areas

To investigate further metabolic pathways that FMT for rCDI may impact upon, we performed proton nuclear magnetic resonance spectroscopy (^1^H-NMR) on stool (fecal water) and urine from rCDI patients participating in a clinical trial comparing capsule to colonoscopic delivery of FMT, with samples collected prior to FMT, and at weeks 1, 4, and 12 post-FMT.^38^ Samples from 18 patients and 3 donors were analyzed; further details on methodology are provided in the **Supplementary Material**.^1^H-NMR is an ‘information-rich’ analytical technique used in metabonomics and popular for its capability to simultaneously detect many biochemical entities (host and microbial) from complex biofluids in a nondestructive manner.^39^

**Figure 1.**
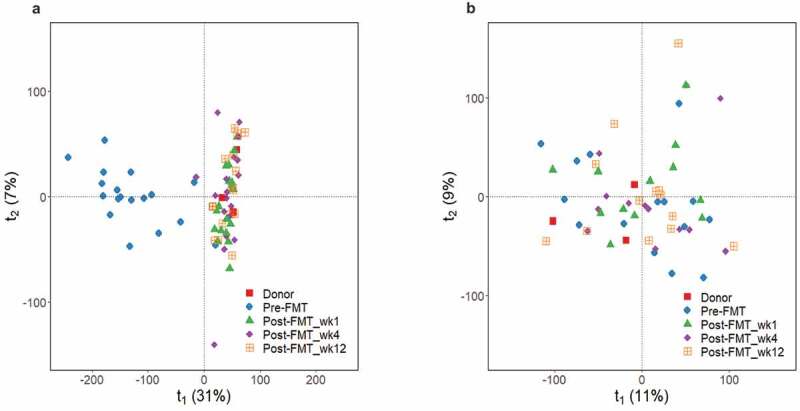
Metabolic profile differences in donors and recipients before and after FMT. Principal component analysis (PCA) scores plots of^1^H-NMR spectra from fecal water (a) and urine (b) samples from donors (*n* = 3) and recipients collected at different timepoints (for stool recipient samples: *n* = 18 for timepoints 0 (pre-FMT), 1, 4 and *n* = 16 for timepoint 12; for urine: *n* = 15 for timepoint 0, *n* = 12 for timepoint 1, *n* = 13 for timepoints 4 and 12).

Global metabolic profiling of fecal water changed notably after FMT, with pre-FMT samples clustering apart from post-FMT and donor samples across the first principal component in the principal components analysis (PCA) scores plot (Figure 1). This indicates that patients acquired a stool metabolic profile more similar to donors already in the first week post-FMT, and this was maintained for 12 weeks. We did not observe any strong effect of the FMT mode on the metabolic profile (**Supplementary Figure 1a**). To assess the proportion of features changing after FMT, we used mixed effects models^40^ with time as categorical variable and using our first time point (pre-FMT) as reference category. Specifically, we tested the time effect as a likelihood ratio test among two models – with or without time as the predictor – fitted to each NMR feature (binned spectra intensities): NMR feature ~ time + FMT mode + (1|Donor) + (1|Recipient). A total of 15,162 out of 21,252 (≈71%) features from fecal water changed with time, which agrees with the clear clustering already observed in the PCA (Figure 1(a)). Using statistical total correlation spectroscopy (STOCSY),^41^ 2D J-resolved (JRES)^1^H-NMR spectra and NMR peak databases, we annotated different metabolites and calculated the area under the curve (AUC) of a representative peak for each, using spectra normalized by probabilistic quotient normalization (PQN).^42^

**Figure 2.**
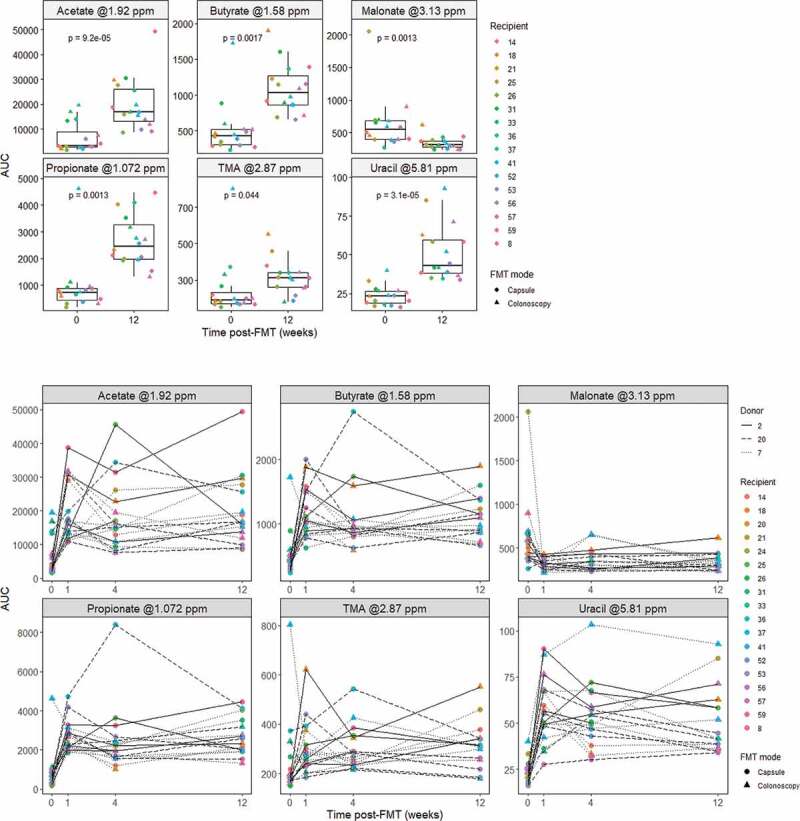
Metabolite changes in fecal water. Area under curve (AUC) of metabolite representative peaks at 0 (pre-FMT) and 12 weeks post-FMT (top; *n* = 18 for timepoint 0 and *n* = 16 for timepoint 12), and across all measured time points (bottom; *n* = 18 for timepoints 0, 1, 4 and *n* = 16 for timepoint 12). *P*-values were calculated using paired Wilcoxon signed rank test.

In stool, the SCFAs acetate, butyrate and propionate increased 4.96, 2.46 and 3.46 median fold at 12 weeks post-FMT, respectively (Figure 2), consistent with previous findings, as described above. Further analysis and interpretation regarding the effect of FMT upon SCFAs (as well as upon bile acids) is provided within the **Supplementary Material**. Interestingly, we also detected a 1.7-fold increase in the microbial metabolite trimethylamine (TMA; produced by gut bacteria from dietary choline and L-carnitine), a 1.95-fold increase in the pyrimidine uracil, and a 0.55-fold decrease in the carboxylic acid malonate (Figure 2). To our knowledge, this is the first time an increase in TMA is reported following FMT intervention in patients with rCDI. Similarly, an increase in uracil has not been reported previously, although a decrease in this metabolite has been observed in feces from mice treated with vancomycin;^43^ as such, the observed uracil increase could be to at least partly a proxy of gut ecology recovery after cessation of vancomycin, rather than an effect of FMT *per se*. The decrease in malonate after FMT has also not been described before. The capacity to metabolize malonate by the gut microbiota and its impact on host metabolism has not previously been characterized in detail, but appears to be negatively-correlated with acetate across different experimental landscapes,^44,45^ and could be associated with an increase in gut bacteria using this metabolite as energy source.

**Figure 3.**
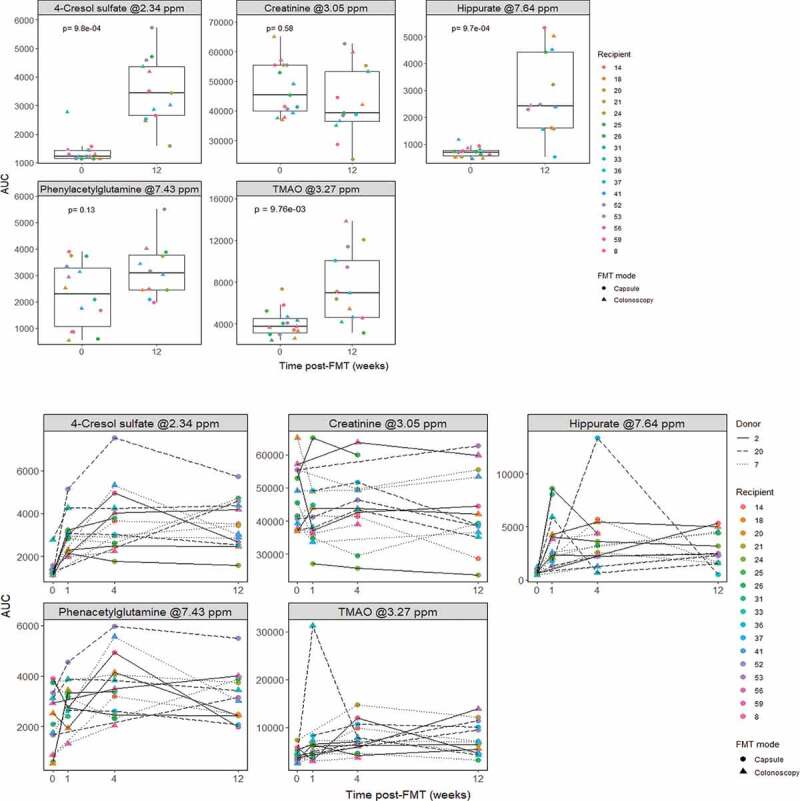
Metabolite changes in urine. AUC of metabolite representative peaks at 0 (pre-FMT) and 12 weeks post-FMT (top; *n* = 15 for timepoint 0 and *n* = 13 for timepoint 12), and across all measured time points (bottom; *n* = 15 for timepoint 0, *n* = 12 for timepoint 1, *n* = 13 for timepoints 4 and 12). *P*-values were calculated using paired Wilcoxon signed rank test.

In urine, PCA scores did not show clear clusters among different groups or FMT modes (Figure 1(b) and **Supplementary Figure 1b**), but 3,099 out of 27,271 features (≈11%) changed with time. Particularly, we found a 3.46-fold increase in the microbial-host co-metabolite hippurate (produced by conjugation of the microbial compound benzoate with glycine in the liver), a 1.85-fold increase in the co-metabolite trimethylamine-N-oxide (TMAO; resulting from TMA oxidation in the liver), and a 2.8-fold increase in 4-cresol sulfate (4-CS; the hepatic sulfonation of microbial 4-cresol). Finally, we also found a 1.34-fold increase in phenylacetylglutamine (PAG) (although this was not significant at the 5% significance level), while no differences were found in creatinine (Figure 3). As with fecal uracil, the observed increases in hippurate and PAG could reflect recovery of gut microbial ecology after vancomycin cessation, as those two metabolites were also reduced in urine from mice treated with vancomycin.^43^

The increase in urinary TMAO correlates with the observed increase in fecal TMA (Figure 2), suggesting a higher availability of TMA for oxidation in the liver. This could also be a consequence of microbial restoration after FMT, as the previous study shows an increase in urine TMAO in control mice as compared to those treated with antibiotic. TMA and TMAO synthesis is markedly altered after antibiotic treatment in humans, with gut microbial production of TMA from L-carnitine appearing to involve multiple commensal bacteria (only partially identified) rather than a single community member.^46^ However, given the association of TMAO with atherosclerosis,^47^ long-term effects of FMT on TMA and TMAO levels in recipient patients deserves further investigation, as well as potentially the need to triage donors according to their TMA or TMAO levels; this is particularly of relevance given the interest of the potential role of FMT in treating obesity/metabolic syndrome (MetS). Given that a vegan diet is associated with reduced capacity to synthesize TMAO on the so-called ‘carnitine challenge’^47^ Smits and colleagues performed a pilot study where 20 male MetS patients were randomized to either receive lean vegan donor FMT or autologous FMT.^48^ Interestingly, while certain gut microbiota changes were seen in the vegan donor FMT recipients, there was neither an improvement of arterial wall inflammation, nor a change in TMA/TMAO metabolism, by two weeks post-FMT.^48^ The value of vegan FMT donors for MetS treatment – together with patient diet and exercise patterns post-FMT – needs to be further evaluated in larger cohorts, and its impact on TMA/TMAO metabolism and associated gut microbes further explored using ‘confounder-reduced’ batch cultures and/or mouse models. Additionally, a clinically-applicable means of assessing the ability of the gut microbiota to produce TMAO – through the oral carnitine challenge test^49^ – may be a useful test for screening of donors in the future.

The increase in 4-CS post-FMT is somewhat less expected, given that 4-cresol is produced by *C. difficile*.^50^ 4-CS has been associated with inflammation and a range of diseases, including kidney failure, autism, and colorectal cancer.^51,52^ The lack of change in creatinine levels after FMT (Figure 3) indicates that kidney function does not appear to be affected by the intervention. As such, one possible explanation may be that FMT disrupts the life cycle of *C. difficile* without necessarily causing its clearance from the gut. Another explanation for the increase in urinary 4-CS is that it may reflect increased bacterial production of 4-cresol after FMT, restored by any of the number of non-*C. difficile* gut bacteria which also produce this metabolite,^53^ with the possible concomitant shift toward increased consumption of proteins as the patient symptoms improve. This would provide the gut microbiota higher amounts of amino acid tyrosine, the 4-cresol substrate. However, detailed dietary data in our study was not available, which is a limitation of this study when interpreting changes in TMAO and 4-cresol. As with TMAO, the long-term impact of FMT on 4-CS levels, and possible causes for its increase other than rCDI – or whether it relates to a higher risk of recurrence – remains to be assessed.

## 4. Conclusions

Our past work and the novel results presented in this *Addendum* suggest a strong impact of FMT on the metabolic profile of recipients soon after intervention. Mechanistic insights using mouse models, chemostats or human samples on the role of these metabolites during rCDI could give us further insight into the pathogenesis of rCDI, biomarkers of FMT outcomes, and – of greatest clinical relevance – the potential to replace FMT with a more refined microbial therapeutic, e.g. a ‘cocktail’ of purified BSH and glycerol trivalerate. Furthermore, consequences of such metabolic shifts, especially at later follow-up time points and controlling for dietary factors, need further investigation, and may be of particular significance as FMT usage extends beyond CDI and into other diseases.

## Supplementary Material

Supplemental Material

## Addendum to:

McDonald J.A.K., Mullish B.H., Pechlivanis A., Liu Z., Brignardello J., Kao D., Holmes E., Li J.V., Clarke T.B., Thursz M.R., Marchesi J.R. Inhibiting growth of *Clostridioides difficile* by restoring valerate, produced by the intestinal microbiota. *Gastroenterology* (2018); 155(5):1495-1507.e15

Monaghan T., Mullish B.H., Patterson J., Wong G.K., Marchesi J.R., Xu H., Jilani T., Kao D. Effective fecal microbiota transplantation for recurrent *Clostridioides difficile* infection in humans is associated with increased signalling in the bile acid-farnesoid X receptor-fibroblast growth factor pathway. *Gut Microbes* (2019);10(2):142-148. doi: 10.1080/19490976.2018.1506667.

Mullish B.H., McDonald J.A.K., Pechlivanis A., Allegretti J.R., Kao D., Barker G.B., Kapila D., Petrof D., Joyce S.A., Gahan G.M., Glegola-Madesjska I., Williams H.R.T., Holmes E., Clarke T.B., Thursz M.R, Marchesi J.R. Microbial bile salt hydrolases mediate the efficacy of faecal microbiota transplant in the treatment of recurrent *Clostridioides difficile* infection. *Gut* (2019); 68(10):1791-1800. doi: 10.1136/gutjnl-2018-317842
