# Supplementary material for: Understanding the mechanisms of efficacy of fecal microbiota transplant in treating recurrent Clostridioides difficile infection and beyond: the contribution of gut microbial-derived metabolites
Source: Gut Microbes. 2020 Sep 6;12(1):1810531. doi: 10.1080/19490976.2020.1810531 (PMC7524310; doi:10.1080/19490976.2020.1810531)
Supplement: Supplemental Material [file KGMI_A_1810531_SM1979.docx]

**Supplementary Material:**

**Understanding the mechanisms of efficacy of fecal microbiota transplant in treating recurrent *Clostridioides difficile* infection and beyond: the contribution of gut microbial-derived metabolites.**

1. **Further methods for proton nuclear magnetic resonance spectroscopy analysis:**

Collection and analysis of human samples for this study received approval from the UK National Research Ethics Centres (13/LO/1867) and Research and Development/Research Ethics Board approval from all collaborating institutions. Paired urine and stool samples were analysed from rCDI patients from a Canadian randomised controlled trial investigating capsulised *vs* colonoscopic FMT as rCDI treatment (18 participants; samples collected pre- and at 1, 4 and 12 weeks post-FMT), together with donors (three participants)^1^. While the clinical aspect of this study had included 116 participants, full availability of serial paired samples was only available for 43 patients, and 18 of these were randomly chosen (9 receiving capsule FMT, 9 receiving colonoscopic FMT) for the analyses performed within this manuscript. All urine and stool samples for metabonomic analysis were collected and kept frozen until processing for analysis, consistent with best practice protocols^2–4^.

Stool samples for two recipients at the 12-week post-FMT timepoint were too limited for analysis, as well as two, five, three and four urine samples from post-FMT timepoints 0, 1, 4 and 12 weeks, respectively. In addition, after quality checks of the spectra (manual and PCA inspection), two urine samples were removed prior to analysis as well as samples from a recipient with very high intensity glucose resonances in urine.

Statistical analyses were performed in R except for the initial spectral binning and STOCSY, for which in-house MATLAB scripts were used. PCA was done using *ropls* package^5^, *lme4* package was used for the linear mixed models, and figures were generated with *ggplot2* and *ggpubr* packages.

1. **Additional analysis and discussion regarding the impact of fecal microbiota transplant for recurrent *C. difficile* infection upon short chain fatty acid metabolism:**

**2.1. Overview:**

While ^1^H-NMR is a sensitive and specific modality for the analysis of short chain fatty acids (SCFAs), the reference standard for their detection and quantification in biofluids is gas chromatography linked to mass spectrometry (GC-MS). Given the clear impact of fecal microbiota transplant (FMT) for recurrent *C. difficile* infection (rCDI) upon SCFA metabolism – and the interest upon the multi-systemic effects of SCFAs within humans and other mammals – we explored this further by performing GC-MS upon matched stool, urine and serum samples from the patients described in **Section 3** of the main *Addendum* manuscript.

Targeted GC MS for SCFA detection, identification and quantification was performed using adaptation of previously-described protocols for the analysis of serum and urine^6^ and for stool^7^ samples. Sample analysis was performed on an Agilent 7890B GC system coupled to an Agilent 5977A mass selective detector (Agilent, Santa Clara, California). Analysis of data was performed using MassHunter software (Agilent), with SCFA concentrations being integrated from a freshly prepared calibration curve. Owing to differences in limits of detection of different SCFAs in different biofluids, not all SCFAs could be quantified in all three biofluids.

- 1. **Fecal SCFA:**

Results for the analysis of the effect of FMT for rCDI upon stool levels of SCFAs are displayed in **Supplementary Figure 2,** extending upon the data regarding for stool valerate alone presented in our earlier work^8^. The levels of all detected SCFAs in feces were significantly lower in patients with rCDI pre-FMT than in the stool of healthy donors (*p*<0.01, Mann-Whitney U). Furthermore, successful FMT for rCDI was associated with rapid and sustained restoration of SCFAs to levels comparable to that of donors (*p*<0.01, Friedman test with Benjamini-Hochberg FDR), including the restoration of gut valerate levels (**Supplementary Figure 2D**)^8^.

- 1. **Serum SCFA:**

Results for the analysis of the effect of FMT for rCDI upon serum levels of SCFAs are presented in **Supplementary Figure 3** (N.B. no serum samples were collected at week 1 post-FMT). Successful FMT was associated with sustained increases in serum levels of butyrate, 2-methylbutyrate, isovalerate and valerate (**Supplementary Figure 3D-G;** *p* <0.05, Friedman test with Benjamini-Hochberg FDR). Whilst there was a trend towards reduced serum levels of a number of SCFAs in pre-FMT samples compared to healthy donors, this only reached statistical significance for 2-methylbutyrate (**Supplementary Figure 3E**; *p* <0.01, Mann-Whitney U test) and valerate (**Supplementary Figure 3G**; *p* <0.01, Mann-Whitney U test).

Previous work by our group, presented in abstract form^9^, has investigated the potential implications of these changes in serum SCFA associated with FMT for rCDI upon the gut-brain axis. This is of interest, since there is evidence that successful FMT for rCDI is associated with long-term improvements in metrics related to mental health and quality of life that cannot be justifiably explained by resolution of CDI symptoms alone^9,10^, and microbiota-mediated mechanisms have been proposed^11^. In these experiments, it was demonstrated that a mixture of SCFAs found at relative concentrations to that of the serum of pre-FMT rCDI patients did not alter the release of pro-inflammatory cytokines from a model of primary microglia from rat brain treated with a pro-inflammatory stimulus (specifically, bacterial lipopolysaccharide or interferon-γ). However, of particular interest, pre-treatment of the model with a mixture of SCFAs found at relative concentrations to that of the serum of successfully-treated post-FMT rCDI patients resulted in a significant reduction in inflammatory mediator increase, including reductions in interleukin-6, tumor necrosis factor and nitric oxide^9^.

- 1. **Urinary SCFA:**

Results for the analysis of the effect of FMT for rCDI upon urinary levels of SCFAs are demonstrated in **Supplementary Figure 4**. FMT appeared to have almost no impact on the urinary levels of almost all SCFA. However, it was noted that urinary levels of both valerate and caproate were lower in pre-FMT samples than they were in donor urine (*p*<0.05, Mann-Whitney U test). In addition, urinary levels of 2-methylbutyrate were higher at week 1 post-FMT compared to pre-FMT samples (*p* <0.01, Friedman test with Benjamini-Hochberg FDR), but not at later time points.

1. **Additional discussion regarding the impact of fecal microbiota transplant for recurrent *C. difficile* infection upon bile acid metabolism:**

It is of note that ultraperformance liquid-chromatography mass spectrometry-based bile acid profiling (UPLC-MS)^4^ has also been performed for the same stool samples analysed by ^1^H-NMR within this *Addendum.* Results from this have been reported previously^12^. In summary, FMT for rCDI was associated with a rapid and sustained reduction in the stool levels of primary conjugated bile acids (including taurocholic acid), marked increases in the stool levels of secondary bile acids (including deoxycholic acid and lithocholic acid); such results are consistent with other comparable studies^13–16^. A further finding of interest from this UPLC-MS analysis, not previously-reported, was that successful FMT for rCDI is associated with a rapid and sustained reduction in the amount of tauro-β-muricholic acid in stool to a comparable level to that of healthy donors (**Supplementary Figure 5**); the explanation for this observation may be primarily related to the gut BSH functionality restored through FMT. Tauro-β-muricholic acid is of particular interest because this bile acid is an FXR antagonist, and therefore hypothesised to be a key factor contributing to the association between the gut microbiota and FXR signalling seen in mice^17^. However, the extent to which this mechanism may be extrapolated to humans is unclear, because of differences in key aspects of relevant physiology between mice and humans, such as the differences in FGF orthologues present in both, and the fact that tauro-β-muricholic acid is only present at relatively modest levels in the human gut compared to other conjugated bile acids^12^.

1. **Supplementary Figures:**

**Supplementary Figure 1:**


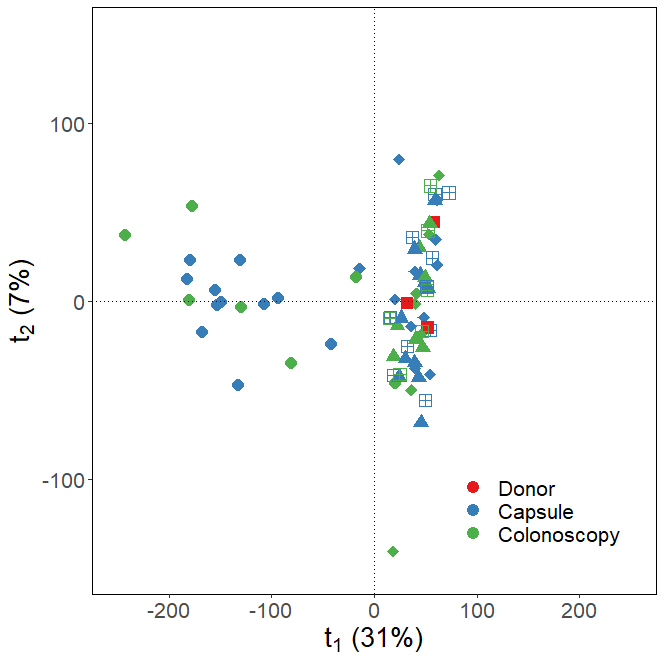


**a**

**b**


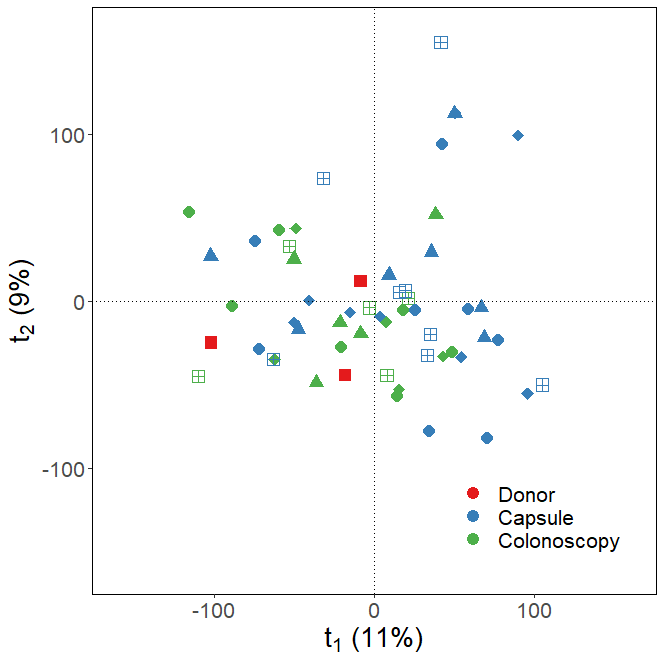


**Supplementary Figure 1: Impact of route of FMT administration (capsule *vs* colonoscopy) upon metabolic profile changes in FMT recipients.** Principal component analysis (PCA) scores plots of ^1^H-NMR spectra from fecal water (a) and urine (b) samples from donors (*n*=3) and recipients collected at different timepoints (for stool recipient samples: *n*=18 for timepoints 0 (pre-FMT), 1, 4 and *n*=16 for timepoint 12; for urine: *n*=15 for timepoint 0, *n*=12 for timepoint 1, *n*=13 for timepoints 4 and 12).

**Supplementary Figure 2:**

**
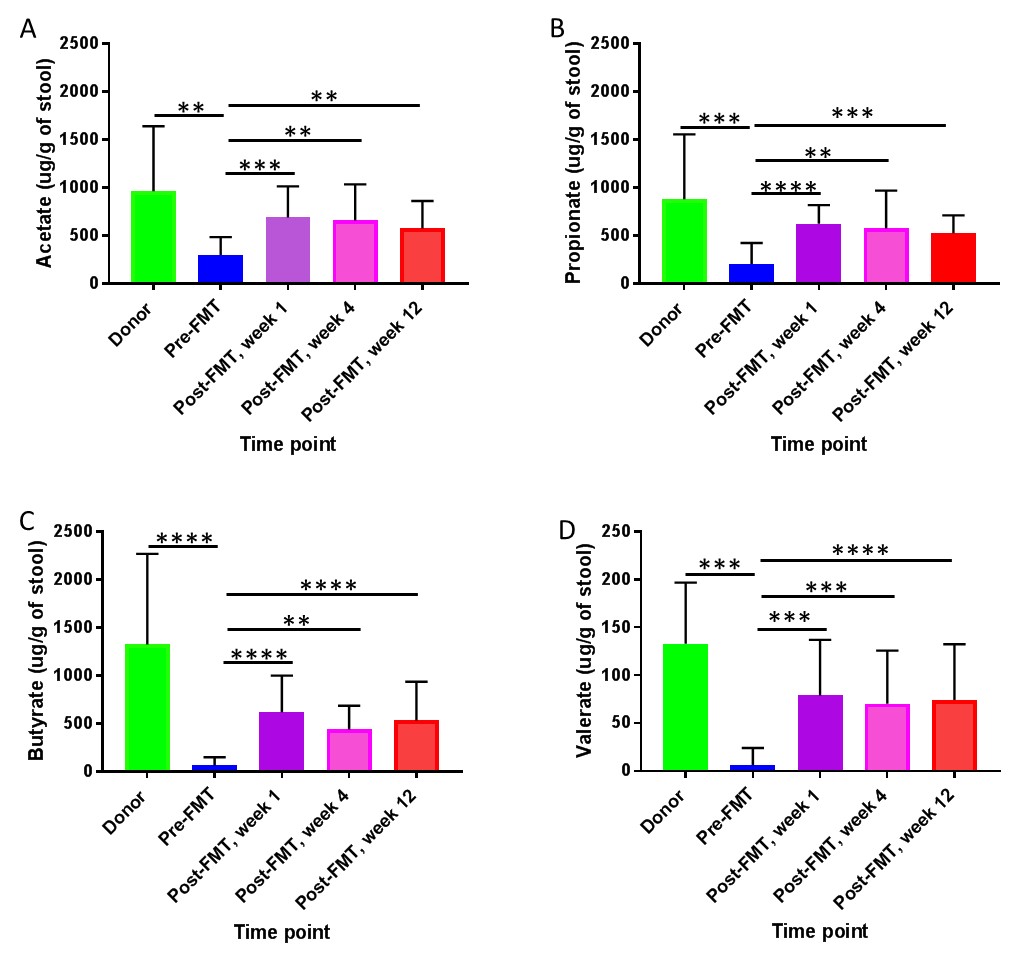
**

**Supplementary Figure 2: Analysis of the effect of FMT for rCDI upon faecal profiles of SCFAs, as assessed using GC-MS.** A: Acetate; B: Propionate; C: Butyrate; D: Valerate (**, *p*<0.01; ***, *p*<0.001; ****, *p*<0.0001; Mann-Whitney U for donor *vs* pre- or post-FMT, Friedman test with Benjamini-Hochberg FDR for pre- *vs* post-FMT) (Donors: *n*=3; rCDI patients pre- and post-FMT: *n*=18). Data presented as mean with standard deviation.

**Supplementary Figure 3:**

**
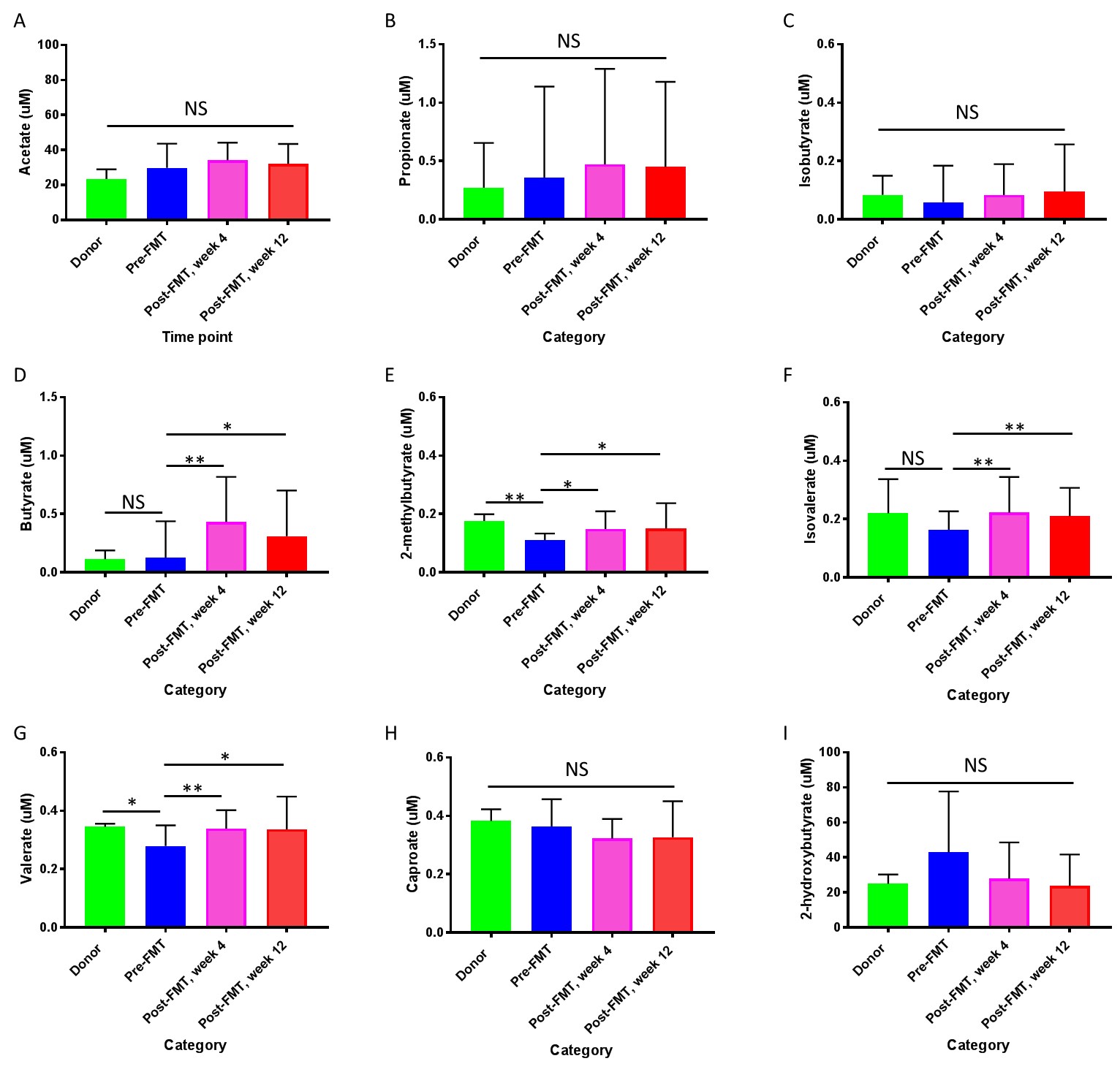
**

**Supplementary Figure 3: Analysis of the effect of FMT for rCDI upon serum profiles of SCFAs, as assessed using GC-MS.** A: Acetate; B: Propionate; C: Isobutyrate; D: Butyrate; E: 2-methylbutyrate; F: Isovalerate; G: Valerate; H: Caproate; I: 2-hydroxybutyrate (*, *p*<0.05; **, *p*<0.01; Mann-Whitney U for donor *vs* pre- or post-FMT, Friedman test with Benjamini-Hochberg FDR for pre- *vs* post-FMT). (Donors: *n*=3; rCDI patients pre- and post-FMT: *n*=18). Data presented as mean with standard deviation.


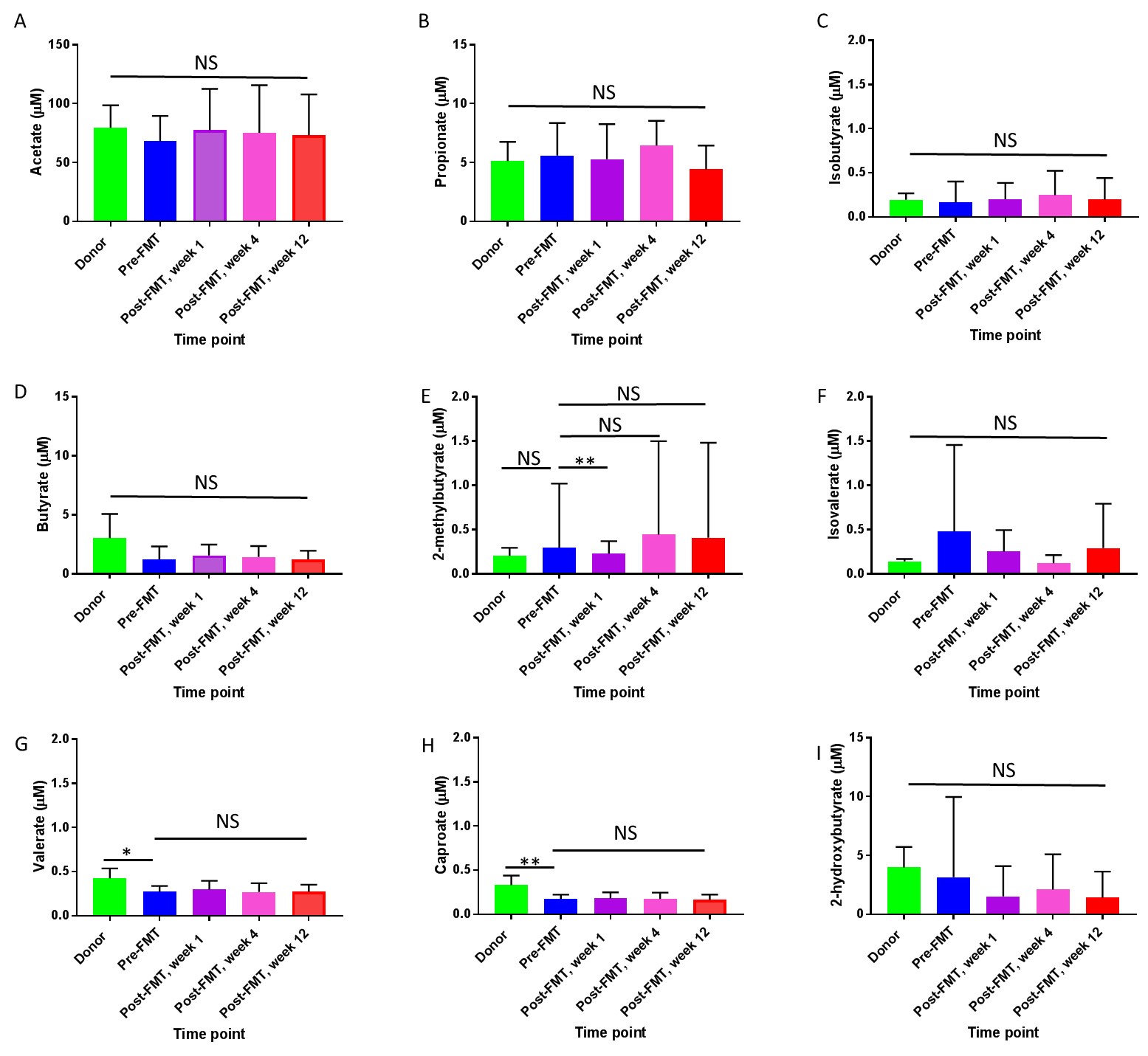
**Supplementary Figure 4:**

**Supplementary Figure 4: Analysis of the effect of FMT for rCDI upon urinary profiles of SCFAs, as assessed using GC-MS.** A: Acetate; B: Propionate; C: Isobutyrate; D: Butyrate; E: 2-methylbutyrate; F: Isovalerate; G: Valerate; H: Caproate; I: 2-hydroxybutyrate (*, *p*<0.05; **, *p*<0.01; Mann-Whitney U for donor *vs* pre- or post-FMT, Friedman test with Benjamini-Hochberg FDR for pre- *vs* post-FMT). (Donors: *n*=3; rCDI patients pre- and post-FMT: *n*=18). Data presented as mean with standard deviation.

**Supplementary Figure 5:**


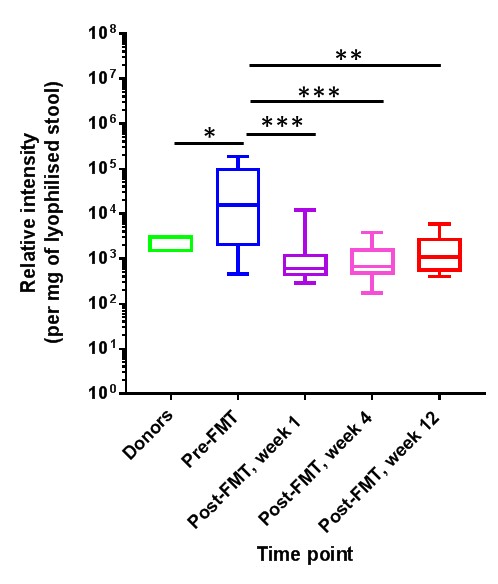


**Supplementary Figure 5: The effect of FMT for rCDI upon fecal tauro-β-muricholic acid levels.** As assessed using UPLC-MS bile acid profiling data (*, *p*<0.05; **, *p*<0.01; ***, *p*<0.001; Mann-Whitney U for donor *vs* pre- or post-FMT, Friedman test with Benjamini-Hochberg FDR for pre- *vs* post-FMT). (Donors: *n*=3; rCDI patients pre- and post-FMT: *n*=18). Centre of box: median; box hinges: 25 – 75% percentile; whiskers: minimum to maximum data points.

**References:**

1. Kao D, Roach B, Silva M, Beck P, Rioux K, Kaplan GG, Chang HJ, Coward S, Goodman KJ, Xu H, et al. Effect of oral capsule– vs colonoscopy-delivered fecal microbiota transplantation on recurrent Clostridium difficile infection: A randomized clinical trial. JAMA - J Am Med Assoc [Internet] 2017 [cited 2017 Dec 12]; 318:1985–93. Available from: http://jama.jamanetwork.com/article.aspx?doi=10.1001/jama.2017.17077

2. Sarafian MH, Lewis MR, Pechlivanis A, Ralphs S, McPhail MJW, Patel VC, Dumas ME, Holmes E, Nicholson JK. Bile Acid Profiling and Quantification in Biofluids Using Ultra-Performance Liquid Chromatography Tandem Mass Spectrometry. Anal Chem [Internet] 2015 [cited 2017 Oct 8]; 87:9662–70. Available from: http://www.ncbi.nlm.nih.gov/pubmed/26327313

3. Gratton J, Phetcharaburanin J, Mullish BH, Williams HRT, Thursz M, Nicholson JK, Holmes E, Marchesi JR, Li JV. Optimized Sample Handling Strategy for Metabolic Profiling of Human Feces. Anal Chem 2016; 88.

4. Mullish BH, Pechlivanis A, Barker GF, Thursz MR, Marchesi JR, McDonald JAK. Functional microbiomics: Evaluation of gut microbiota-bile acid metabolism interactions in health and disease [Internet]. Methods2018 [cited 2018 May 2]; 149:49–58. Available from: https://www.sciencedirect.com/science/article/pii/S104620231730422X

5. Thévenot EA, Roux A, Xu Y, Ezan E, Junot C. Analysis of the Human Adult Urinary Metabolome Variations with Age, Body Mass Index, and Gender by Implementing a Comprehensive Workflow for Univariate and OPLS Statistical Analyses. J Proteome Res [Internet] 2015 [cited 2020 Jul 10]; 14:3322–35. Available from: https://pubs.acs.org/sharingguidelines

6. Moreau NM, Goupry SM, Antignac JP, Monteau FJ, Le Bizec BJ, Champ MM, Martin LJ, Dumon HJ. Simultaneous measurement of plasma concentrations and 13C-enrichment of short-chain fatty acids, lactic acid and ketone bodies by gas chromatography coupled to mass spectrometry. J Chromatogr B Anal Technol Biomed Life Sci 2003; 784:395–403.

7. García-Villalba R, Giménez-Bastida JA, García-Conesa MT, Tomás-Barberán FA, Carlos Espín J, Larrosa M. Alternative method for gas chromatography-mass spectrometry analysis of short-chain fatty acids in faecal samples. J Sep Sci [Internet] 2012 [cited 2018 Feb 23]; 35:1906–13. Available from: http://www.ncbi.nlm.nih.gov/pubmed/22865755

8. McDonald JAK, Mullish BH, Pechlivanis A, Liu Z, Brignardello J, Kao D, Holmes E, Li J V., Clarke TB, Thursz MR, et al. Inhibiting Growth of Clostridioides difficile by Restoring Valerate, Produced by the Intestinal Microbiota. Gastroenterology [Internet] 2018; 155:1495-1507.e15. Available from: https://linkinghub.elsevier.com/retrieve/pii/S0016508518347711

9. Churchward MA, Michaud ER, Blanco JM, Garcia-Perez I, Mullish BH, Marchesi J, Xu H, Kao DH, Todd K. Sa1924 – Effect of Short Chain Fatty Acids on Gut-Brain Axis Using a Microglial Cell Model. Gastroenterology 2019; 156:S-455.

10. Jalanka J, Hillamaa A, Satokari R, Mattila E, Anttila V-J, Arkkila P. The long-term effects of faecal microbiota transplantation for gastrointestinal symptoms and general health in patients with recurrent *Clostridium difficile* infection. Aliment Pharmacol Ther [Internet] 2018 [cited 2018 Feb 26]; 47:371–9. Available from: http://doi.wiley.com/10.1111/apt.14443

11. Mullish BH. Letter: improvements in mental health after faecal microbiota transplantation—an underexplored treatment-related benefit? Aliment Pharmacol Ther [Internet] 2018 [cited 2018 May 17]; 47:1562–3. Available from: https://onlinelibrary.wiley.com/doi/abs/10.1111/apt.14626

12. Monaghan T, Mullish BH, Patterson J, Wong GKSK, Marchesi JR, Xu H, Jilani T, Kao D. Effective fecal microbiota transplantation for recurrent Clostridioides difficile infection in humans is associated with increased signalling in the bile acid-farnesoid X receptor-fibroblast growth factor pathway. Gut Microbes [Internet] 2019 [cited 2018 Sep 9]; 10:1–7. Available from: http://www.ncbi.nlm.nih.gov/pubmed/30183484

13. Mullish BH, McDonald JAKK, Pechlivanis A, Allegretti JR, Kao D, Barker GF, Kapila D, Petrof EO, Joyce SA, Gahan CGMM, et al. Microbial bile salt hydrolases mediate the efficacy of faecal microbiota transplant in the treatment of recurrent Clostridioides difficile infection. Gut [Internet] 2019 [cited 2019 Feb 11]; 68:1791–800. Available from: https://gut.bmj.com/content/early/2019/02/11/gutjnl-2018-317842

14. Seekatz AM, Theriot CM, Rao K, Chang Y-M, Freeman AE, Kao JY, Young VB. Restoration of short chain fatty acid and bile acid metabolism following fecal microbiota transplantation in patients with recurrent Clostridium difficile infection. Anaerobe [Internet] 2018 [cited 2019 Mar 4]; 53:64–73. Available from: https://www.sciencedirect.com/science/article/pii/S1075996418300581#bib52

15. Weingarden AR, Chen C, Bobr A, Yao D, Lu Y, Nelson VM, Sadowsky MJ, Khoruts A. Microbiota transplantation restores normal fecal bile acid composition in recurrent *Clostridium difficile* infection. Am J Physiol Liver Physiol 2014; 306:G310–9.

16. Allegretti JR, Kearney S, Li N, Bogart E, Bullock K, Gerber GK, Bry L, Clish CB, Alm E, Korzenik JR. Recurrent Clostridium difficile infection associates with distinct bile acid and microbiome profiles. Aliment Pharmacol Ther [Internet] 2016 [cited 2017 Oct 7]; 43:1142–53. Available from: http://www.ncbi.nlm.nih.gov/pubmed/27086647

17. Sayin SI, Wahlström A, Felin J, Jäntti S, Marschall HU, Bamberg K, Angelin B, Hyötyläinen T, Orešič M, Bäckhed F. Gut microbiota regulates bile acid metabolism by reducing the levels of tauro-beta-muricholic acid, a naturally occurring FXR antagonist. Cell Metab [Internet] 2013 [cited 2018 Jan 16]; 17:225–35. Available from: http://www.ncbi.nlm.nih.gov/pubmed/23395169
